# Zolbetuximab-related gastritis: a case report of the patient with prolonged gastrointestinal symptoms

**DOI:** 10.1007/s10120-025-01607-9

**Published:** 2025-04-03

**Authors:** Yuya Sugiyama, Hiroki Tanabe, Shion Tachibana, Kohei Iribe, Sayaka Yuzawa, Hiroyuki Iwaki, Yukinori Yoshida, Mikihiro Fujiya

**Affiliations:** 1 Department of Gastroenterology, Sunagawa City Hospital, Sunagawa, Hokkaido Japan; 2 Division of Gastroenterology, Department of Medicine, Asahikawa Medical University, Asahikawa, Hokkaido Japan; 3 Oncology Center, Asahikawa Medical University Hospital, Asahikawa, Hokkaido Japan; 4 Department of Diagnostic Pathology, Asahikawa Medical University Hospital, Asahikawa, Hokkaido Japan; 5 Department of Diagnostic Pathology, Sunagawa City Hospital, Sunagawa, Hokkaido Japan

**Keywords:** Zolbetuximab, Gastritis, Gastric cancer

## Abstract

A 73-year-old male patient presented with anemia and was diagnosed with unresectable advanced gastric cancer with distant lymph node metastases. The biopsy specimen showed a poorly differentiated adenocarcinoma. Immunohistochemistry was negative for human epidermal growth factor receptor 2, positive for claudin- 18, and revealed a preserved mismatch repair status. A regimen of capecitabine, oxaliplatin, and zolbetuximab was chosen as the primary chemotherapy regimen. On day 2, the patient started complaining of nausea and decreased appetite, and his symptoms gradually worsened. Esophagogastroduodenoscopy performed on day 11 revealed an erythematous and edematous mucosa with white secretions throughout the stomach. A histopathological examination revealed epithalaxia at the surface and severe inflammatory cell infiltration in the lamina propria. These endoscopic and histological findings indicated zolbetuximab-related gastritis. His symptoms improved three weeks after the discontinuation of chemotherapy. Endoscopic and pathological improvements of the gastritis were confirmed three months after the discontinuation of zolbetuximab. This report describes the first case of prolonged severe gastrointestinal symptoms with severe gastritis caused by zolbetuximab, as demonstrated by endoscopic and histopathological evidence.

**Supplementary Information:**

The online version contains supplementary material available at 10.1007/s10120-025-01607-9.

## Introduction

Zolbetuximab, a chimeric (mouse/human IgG_1_) monoclonal antibody targeting claudin-18 isoform 2 (CLDN18.2), is a novel therapeutic agent for CLDN18.2-positive, human epidermal growth factor receptor 2 (HER2)-negative locally advanced unresectable or metastatic gastric or gastroesophageal junction adenocarcinoma. Two phase 3 trials (SPOTLIGHT and GLOW trials) showed that zolbetuximab significantly improved progression-free survival (PFS) and overall survival (OS) when combined with mFOLFOX6 (fluorouracil + leucovorin + oxaliplatin) or CAPOX (capecitabine + oxaliplatin) relative to placebo combined with mFOLFOX6 or CAPOX [1, 2]. The two trials showed that zolbetuximab frequently induced gastrointestinal adverse events (AEs) such as nausea, vomiting, and decreased appetite. Any-grade AEs were observed in more than half of the cases, and grade ≥ 3 nausea and vomiting were observed in approximately 20% of the cases. Nausea and vomiting were observed within the first hour of the initial zolbetuximab infusion [1, 2]. Moreover, all gastrointestinal AEs were transient, and no cases of prolonged AEs have been reported. The detailed mechanisms underlying gastrointestinal adverse events remain unclear.

We report a case of severe gastrointestinal symptoms of prolonged nausea and decreased appetite, with gastritis after administration of zolbetuximab based on endoscopic and histopathological evidence.

## Case presentation

**Fig. 1:**
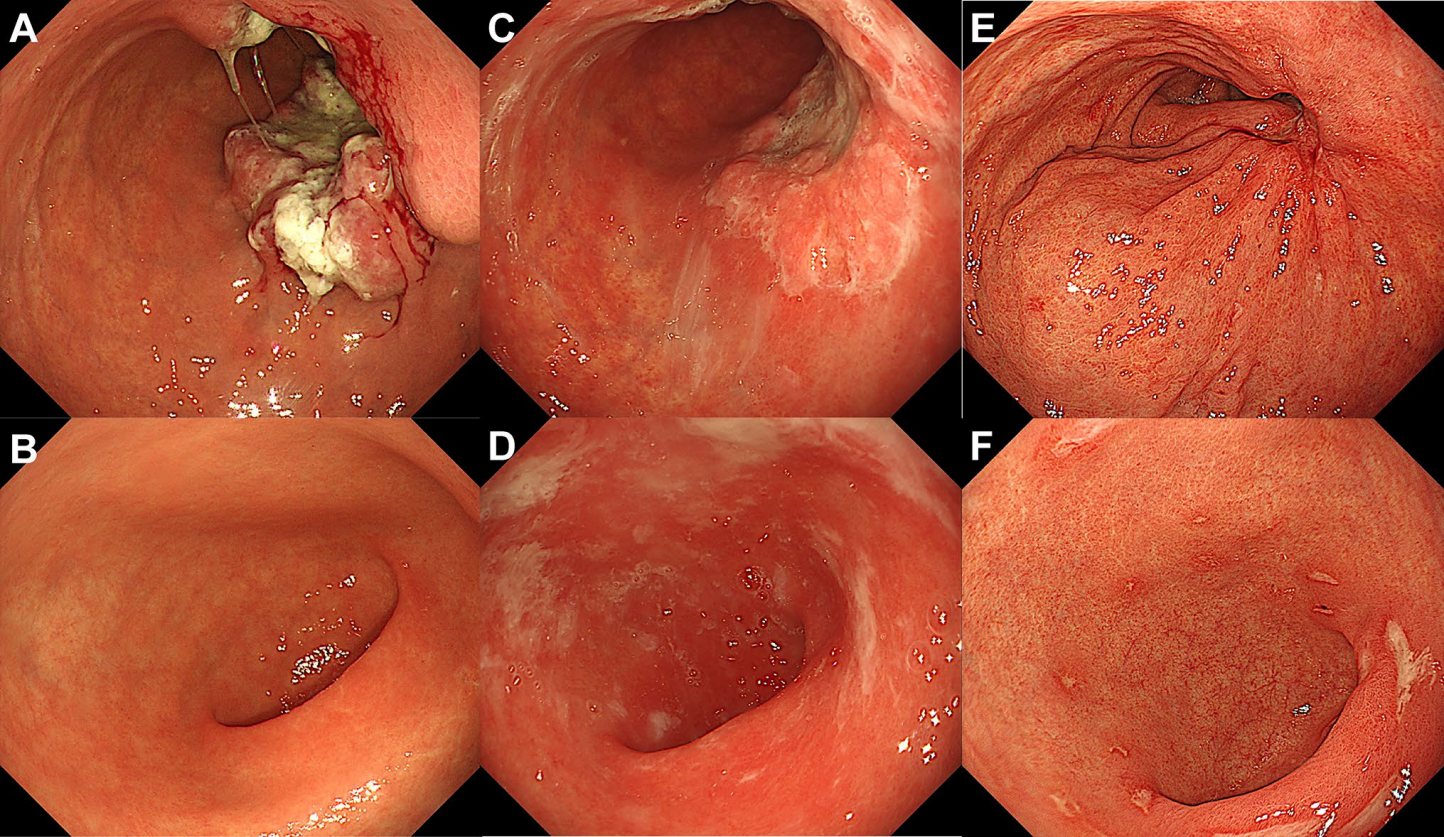
The endoscopic findings before and after the administration of zolbetuximab. Pretreatment (**A**, **B**), 11 days after administration (**C**, **D**) and 3 months after the discontinuation of zolbetuximab (**E**, **F**). A semi-circumferential Type 2 tumor (**A**) is shown in the lesser curvature of the gastric corpus against a background of atrophic gastric mucosa (**B**). The gastric carcinoma shrunk (**C**), and an erythematous and edematous mucosa with white secretions was observed throughout the whole stomach (**D**). Remarkable tumor shrinkage is evident (**E**), and inflammatory changes improved throughout the stomach (**F**)

**Fig. 2:**
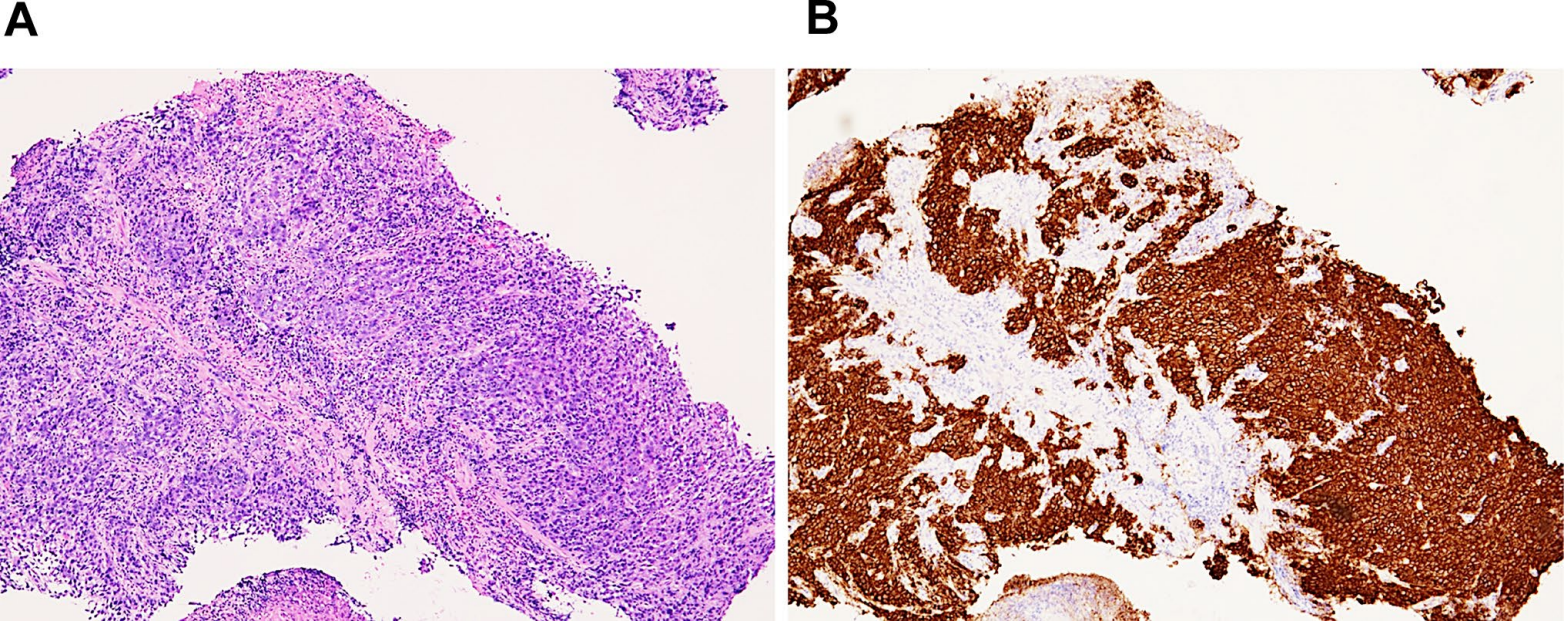
Histopathological findings at the tumor sites. **A** The histological type of the tumor is poorly differentiated carcinoma (H&E, × 100). **B** Tumor cells are claudin 18-positive (> 75%). Used antibody was VENTANA OptiView CLDN18 (43-14A; Roche Diagnostics, Tokyo, Japan) (×100)

**Fig. 3:**
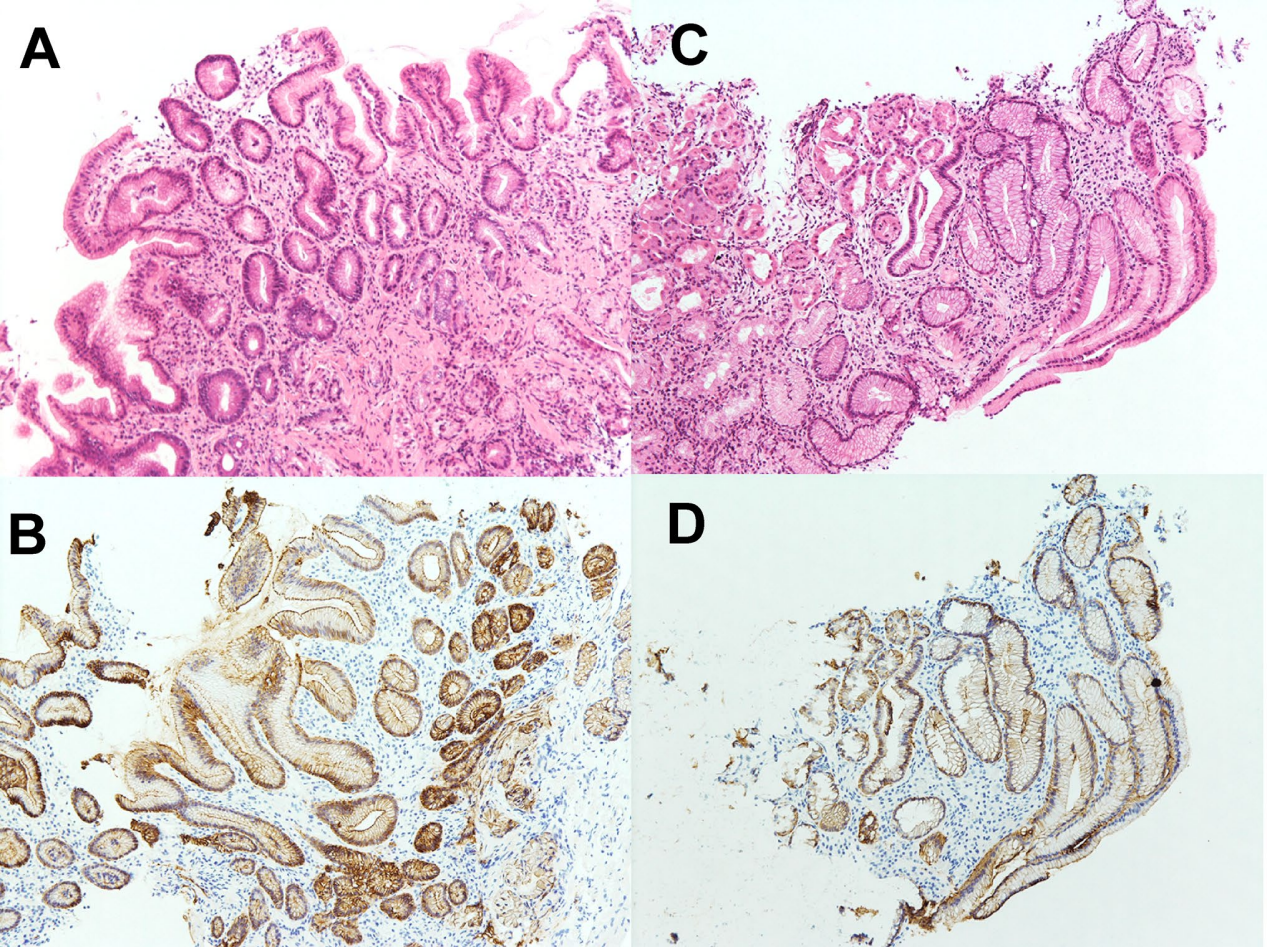
Pre-treatment histopathological findings of non-tumor sites in the stomach. **A**, **B** corpus; **C**, **D** antrum. **A** Biopsy specimen of non-tumor sites in the gastric corpus shows mild atrophic gastritis with mild inflammatory cell infiltration and a preserved glandular duct structure (H&E, ×100). **B** Gastric foveolar epithelia and glandular cells are claudin 18-positive (×100). Claudin18 antibody (34H14L15; abcam, Cambridge, UK) was used for the immunohistochemistry. **C** Non-tumor sites in the antrum show mild gastritis with inflammatory cell infiltration (H&E, ×100). **D** Gastric epithelial cells are claudin 18-positive (×100)

**Fig. 4:**
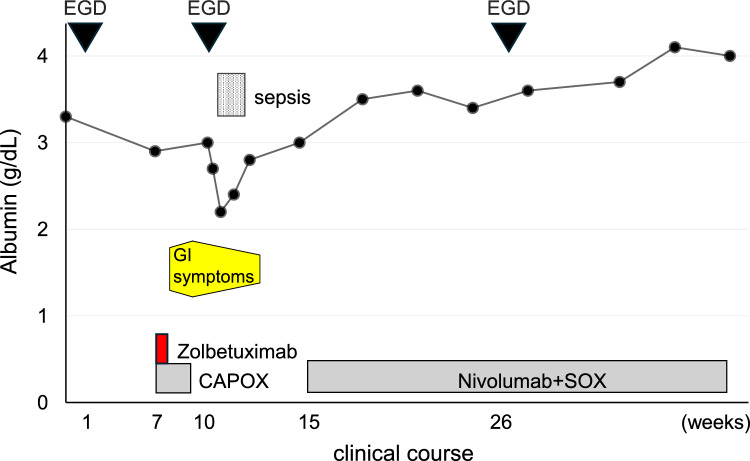
Clinical course and serum albumin level of the patient. Gastrointestinal symptom including nausea, decreased appetite, and heartburn developed on day 2, gradually worsened over time, and continued for more than a month. EGD: esophagogastroduodenoscopy, GI: gastrointestinal, CAPOX: capecitabine + oxaliplatin, SOX: tegafur gimeracil oteracil potassium + oxaliplatin

**Fig. 5:**
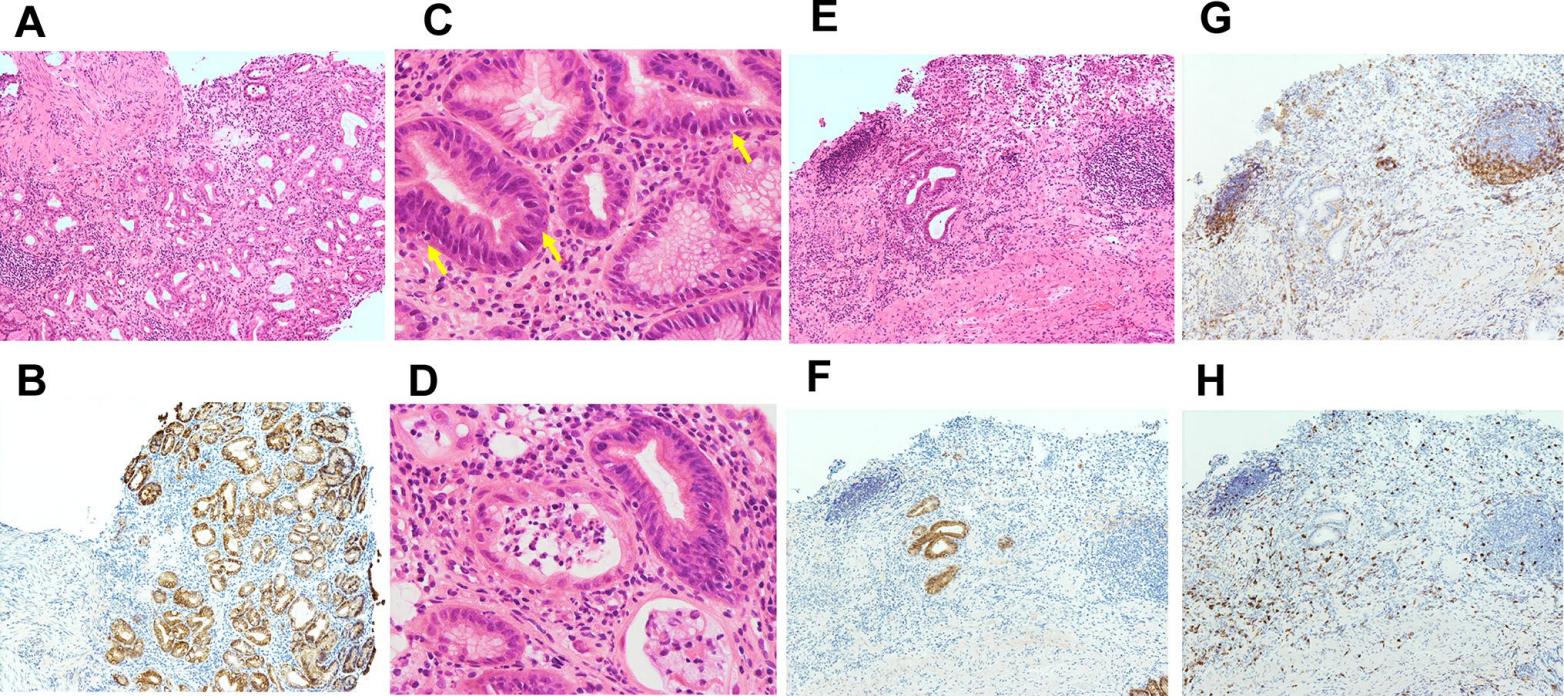
Histopathological findings of zolbetuximab-related gastritis in non-tumor site of the stomach. **A**–**D** corpus; **E**–**H** antrum. **A** Biopsy specimens of the inflammatory lesions in the gastric corpus show erosion at the surface and severe diverse inflammatory cell infiltration in the lamina propria (H&E, ×100). **B** Gastric epithelial cells are specifically claudin 18-positive (×100). Claudin-18 antibody (34H14L15; abcam, Cambridge, UK) was used for the immunohistochemistry. **C** Apoptosis of foveolar epithelium is indicated by yellow arrows (H&E, ×200). **D** Glandular atrophy and dropout are observed (H&E, ×200). **E** Biopsy specimens of the non-inflammatory sites in the gastric antrum show epithalaxia with severe inflammatory cell infiltration (H&E, ×100). **F** Remained glandular cells are stained with the claudin 18 antibody (×100). CD4 lymphocytes (**G**) and CD8 lymphocytes (**H**) are diffusely observed in the inflammatory lesions (×100). Immune subtype markers, including CD4 (1F6; Leica Microsystems, Wetzlar, Germany) and CD8 (C8/144B; Agilent Technology, Santa Cruz, CA, USA) were used for immunohistochemistry

A 73-year-old male patient presented to our department with anemia that was incidentally recognized during exertional angina by the Department of Cardiology in our hospital. A physical examination revealed the following: body height, 165.0 cm; body weight, 67.5 kg; and body mass index, 24.75 kg/m^2^. The patient’s peripheral blood counts were as follows: white blood cell count, 14,900 /µl; hemoglobin, 12.3 g/dl; and platelet count, 35.1 × 10^4^/µl. A laboratory examination revealed elevated inflammatory markers, including a C-reactive protein (CRP) level of 0.47 mg/dL, and hypoalbuminemia with an albumin level of 3.2 g/dL. The patient’s tumor marker levels (CEA and CA19-9) were within the normal range. He was administered low-dose aspirin and vonoprazan, but not nonsteroidal anti-inflammatory drugs (NSAIDs). Esophagogastroduodenoscopy (EGD) revealed a semi-circumferential type 2 tumor in the lesser curvature of the gastric corpus, against a background of atrophic gastric mucosa (Fig. 1A, B). A histopathological examination of the lesion revealed poorly differentiated carcinoma with CLDN18 positivity (Fig. 2A, B), HER2 negativity, preserved mismatch repair status (pMMR), and a programmed cell death-ligand 1 (PD-L1) combined positive score (CPS) of ≥ 10. A histopathological examination of non-tumor sites in the gastric corpus and antrum showed mild inflammatory cell infiltration and preserved glandular duct structures (Fig. 3A–D). Mild atrophy was observed in the gastric foveolar epithelial cells, whose cell surface was positively stained with the CLDN18 antibody. However, intestinal metaplasia was absent. Regarding the *Helicobacter pylori* (HP) infection status, serum HP-IgG was positive, but stool antigen was negative, and a histological examination was negative for HP. Contrast-enhanced computed tomography (CT) revealed irregular wall thickness with a contrast effect in the entire gastric corpus and multiple lymph node metastases (#1, #3a, #4sa, #4d, #5, #6, #7, #8a, #9, #12a, #16a2, and #16b1). Fluorodeoxyglucose positron emission tomography-computed tomography (FDG PET-CT) revealed an abnormal uptake, consistent with the CT findings. These findings confirmed the diagnosis of advanced gastric cancer (T4aN3M1(LYM)). The patient was treated with a CAPOX + zolbetuximab regimen. The infusion rate of zolbetuximab was increased every hour as follows: 50 mg/m^2^/h for the first hour, 100 mg/m^2^/h for the second hour, 150 mg/m^2^/h for the third hour, and 200 mg/m^2^/h for the fourth hour. Multiple antiemetics (dexamethasone, palonosetron, fosnetupitant, and olanzapine) were administered to prevent zolbetuximab-related emesis. No adverse events occurred during or after the infusion of zolbetuximab on day 1. Thereafter, nausea, decreased appetite, and heartburn developed on day 2, and gradually worsened over time (Fig. 4). He was able to ingest fluids and responded well to pre-request antiemetic medications. Therefore, he was followed up on an outpatient basis. However, on day 10, he was unable to ingest fluids and was hospitalized for rehydration. EGD on day 11 revealed tumor shrinkage and an erythematous and edematous mucosa with white secretions throughout the stomach (Fig. 1C, D). A histopathological examination of the inflammatory lesions showed erosion with severe inflammatory cell infiltration in the lamina propria (Fig. 5A–H). Increased apoptosis of the foveolar epithelium and glandular atrophy/dropout were also observed (Fig. 5C, D). Based on these findings, the patient was diagnosed with zolbetuximab-related gastritis. Thereafter, the patient developed septic shock due to a catheter-related bloodstream infection and required intensive treatment. Therefore, chemotherapy was temporarily discontinued. The gastrointestinal symptoms continued for more than a month, until recovery of his general condition. The patient refused the CAPOX + zolbetuximab regimen and selected the secondary chemotherapy with SOX (tegafur gimeracil oteracil potassium + oxaliplatin) + nivolumab regimen. After the introduction of the treatment, the patient had no adverse events. Severe hypoalbuminemia was observed while the septic condition and was gradually improved while the second chemotherapy regimen (Fig. 4). Three months after discontinuation of zolbetuximab, EGD revealed a remarkable improvement in the inflammatory changes in the whole stomach and remarkable tumor shrinkage (Fig. 1E, F). Moreover, histopathological findings showed improvement in inflammatory cell infiltration and regeneration of the glandular epithelium (Supple.Fig. 1A–D).

## Discussion

CLDN18.2, a tight junction protein that is specifically expressed in gastric mucosal cells, is frequently retained in gastric and gastroesophageal junction adenocarcinoma cells. During the process of malignant transformation, the loss of cell polarity may expose CLDN18.2 on the cell surface, making it more accessible to antibodies [3, 4]. Zolbetuximab, a chimeric (mouse/human IgG1) monoclonal antibody targeting CLDN18.2, has been developed for the treatment of gastric and gastroesophageal adenocarcinomas based on these findings, showing promise in clinical trials [1, 2]. While its efficacy in clinical trials is well known, the understanding of its safety profile remains limited.

Frequent adverse events related to zolbetuximab include acute gastrointestinal symptoms, such as nausea, vomiting, and decreased appetite. Moreover, in most cases, symptoms are transient and can be managed by pausing or slowing the infusion of zolbetuximab. Shimozaki et al. reported that zolbetuximab plus chemotherapy could be safely introduced by managing with a proper protocol for initiating zolbetuximab and prophylactic antimetics [5]. We present a rare case of late-onset and prolonged gastrointestinal adverse events associated with zolbetuximab, which were identified as gastritis based on endoscopic and histopathological assessments. To our knowledge, this is the first report indicating a direct relationship between zolbetuximab-related toxicity and gastritis. In our case, the onset of symptoms was delayed, and persistence was notable. We performed EGD to identify the cause of the prolonged gastrointestinal symptoms. Endoscopic findings revealed erythematous and edematous changes with white exudate throughout the entire stomach, and a histopathological analysis demonstrated severe inflammatory cell infiltration, supporting the diagnosis of zolbetuximab-related gastritis. As these findings were not observed before the administration of chemotherapy, it was clear that the patients developed prolonged gastrointestinal symptoms due to the development of severe gastritis after the administration of chemotherapy. The fact that the patient's symptoms did not flare up when nivolumab plus SOX was administered after discontinuing zolbetuximab plus CAPOX further supports our conclusion that zolbetuximab was the causative agent. CLDN18 expression in the normal gastric mucosa was observed at the cell surface of foveolar epithelial cells and glandular cells. Apoptotic bodies in the foveolar epithelium and glandular atrophy/dropout were observed in the gastritis. These epithelial damages are thought to be caused by on-target/off-tumor toxicity, given that they occur more frequently in patients who have not undergone gastrectomy [6].

Little is known about the relationship between zolbetuximab and gastritis; however, Kinugasa et al. reported a study in which ferrets treated with zolbetuximab developed gastritis [7]. After administering zolbetuximab to the ferrets, they were sacrificed and macroscopically and pathologically evaluated. The ferrets developed gastritis, and their inflammation worsened over time. The histopathological examination showed that the surface of the gastric mucosa was the primary site of the damage. Moreover, Hayashi et al. demonstrated that CLDN18-knockdown mice developed gastritis due to paracellular barrier leakage of H + caused by tight junction barrier dysfunction [8]. These experiments have indicated that zolbetuximab can cause gastritis through CLDN18-targeting action. No studies have described endoscopic examinations during zolbetuximab treatment, although adverse events associated with gastrointestinal symptoms are frequently observed in clinical trials [1, 2].

However, the etiology of gastritis should be carefully considered as the treatment options are different [9]. In this case, the patient was receiving low-dose aspirin, but was also receiving vonoprazan and had been taking it prior to the start of chemotherapy. The patient did not receive any other NSAIDs or any new initiating agents. Regarding the HP infection status, serum HP-IgG was positive, stool antigens were negative, and no HP was observed pathologically. In addition, endoscopic findings did not indicate a current infection, suggesting that the patient had been infected with HP in the past. The association between cytotoxic drugs and mucositis is known, and the capecitabine and oxaliplatin administered in this case are also known causative agents. However, chemotherapy-induced mucositis has rarely been reported in the stomach, and it is highly unlikely that capecitabine and oxaliplatin cause gastritis [10, 11]. Recently, it was reported that immune checkpoint inhibitors cause immune-related adverse event (irAE) gastritis. IrAE gastritis shows characteristic endoscopic findings, such as network-pattern erosion and ulcers in the antrum, erythematous and edematous mucosa covered with excessive whitish purulent discharge in the whole stomach, and fragile mucosa [12, 13]. Histopathologically, inflammatory cell infiltration, fibrinopurulent exudate, apoptosis, and decreased gland have been reported. The pathological features in our case suggest that zolbetuximab-related gastritis may be immune-related, similar to irAE gastritis. An accurate diagnosis of such cases of gastritis is crucial and can be achieved through a timely endoscopic examination and biopsy. Zolbetuximab-related AEs are managed with antiemetic medications and infusion-related reactions are treated with premedication including antihistamines and corticosteroids [14]. Therefore, corticosteroid therapy may be effective for zolbetuximab-related gastritis. Prospective studies will be required to clarify the adverse reactions of this drug and the treatment strategy in the future.

In laboratory examinations, the patient showed progressive hypoalbuminemia after the zolbetuximab + CAPOX regimen. We needed to consider the association of this case with Ménétrier’s disease and protein-losing gastroenteropathy. Endoscopic examinations did not show gastric fold hypertrophy, which is a characteristic finding of Ménétrier’s disease. Furthermore, histological analysis did not show dilated lymphatics or foveolar hyperplasia with corkscrew morphology, which are characteristic of protein-losing gastroenteropathy and Ménétrier’s disease, respectively. Therefore, the progressive hypoalbuminemia was likely caused by a reduced intake due to gastrointestinal symptoms and incidentally developed sepsis.

In conclusion, this report provides novel insights into the safety profile of zolbetuximab, with the first case of delayed-onset and persistent gastritis linked to its use. In cases of prolonged gastrointestinal AEs after the administration of zolbetuximab, EGD may lead to the early and crucial diagnosis of zolbetuximab-related gastritis. These findings imply the need for further research into the gastrointestinal effects of zolbetuximab, emphasizing the importance of accumulating more cases in the future.

## Supplementary Information

Below is the link to the electronic supplementary material.

Supplementary file1 (DOCX 45107 KB)

## Data Availability

The datasets generated during and/or analyzed during the current study are available from the corresponding author upon reasonable request.
