# Supplementary material for: Zolbetuximab-related gastritis: a case report of the patient with prolonged gastrointestinal symptoms
Source: Gastric Cancer. 2025 Apr 3;28(4):705–11. doi: 10.1007/s10120-025-01607-9 (PMC12174249; doi:10.1007/s10120-025-01607-9)
Supplement: Supplementary file 1 — Supplementary file1 (DOCX 45107 KB) [file 10120_2025_1607_MOESM1_ESM.docx]

**Supplementary Figure 1.** Post-treatment histopathological findings of non-tumor sites in the stomach. (A, B: corpus; C, D: antrum)


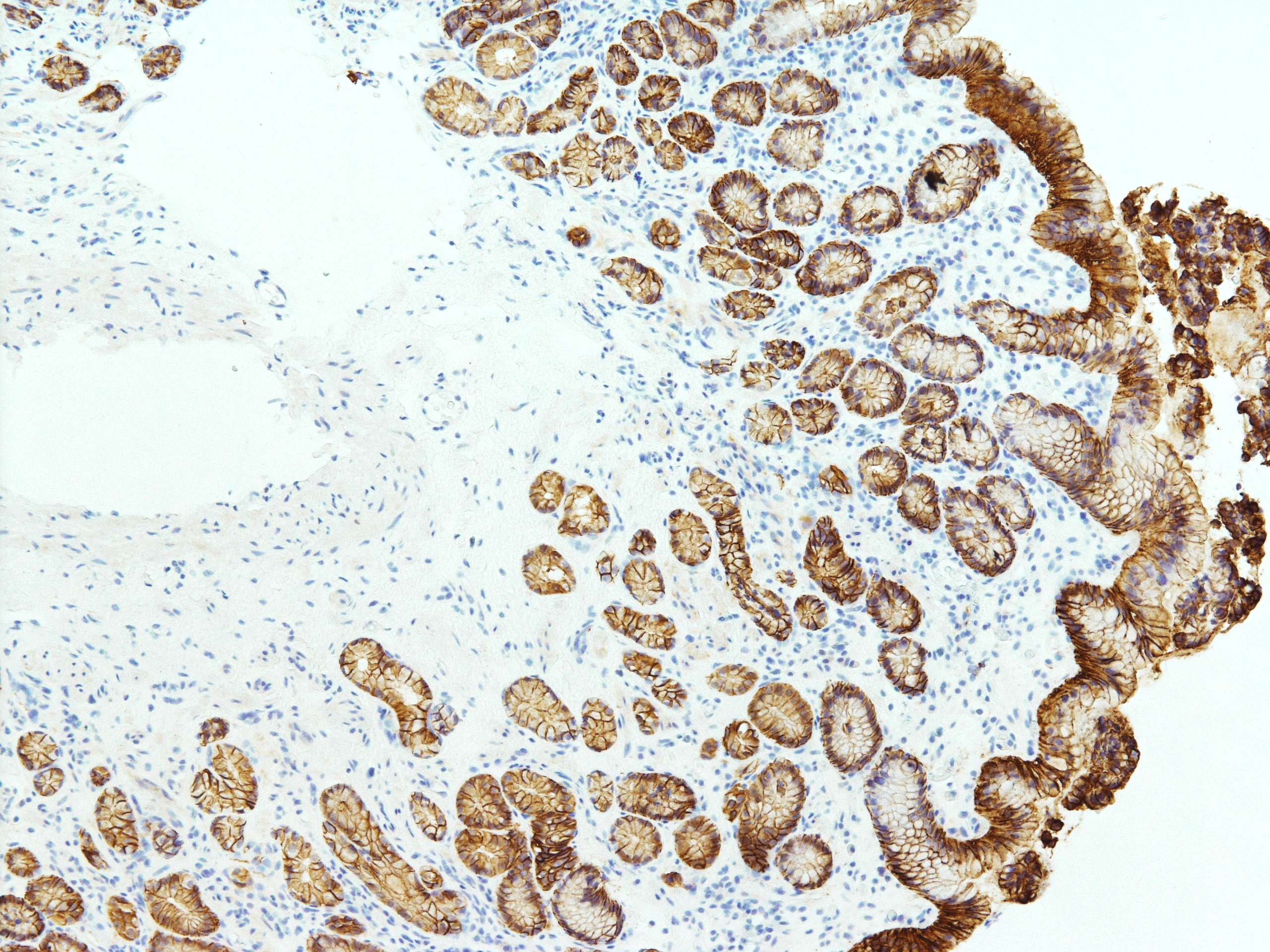

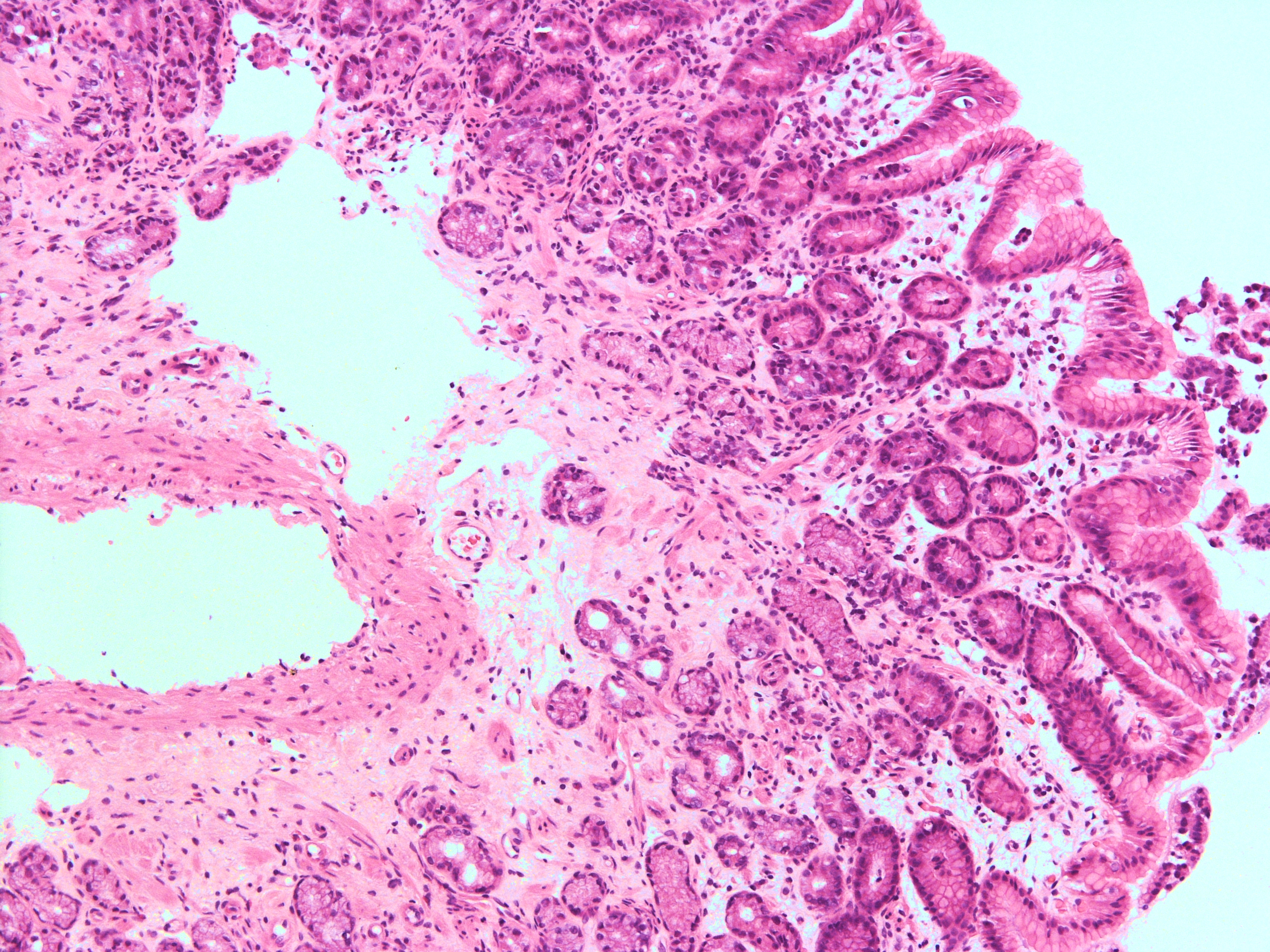

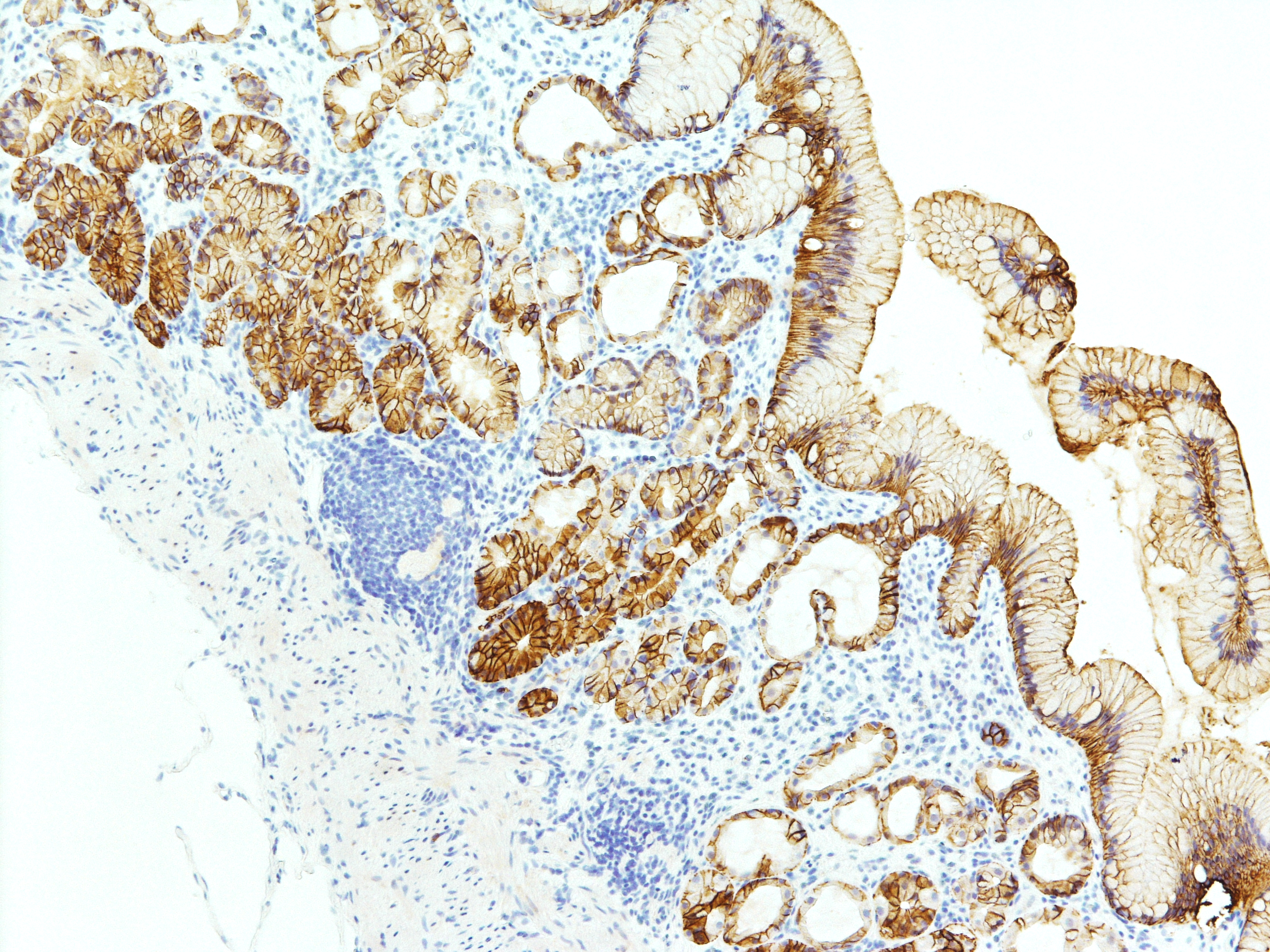

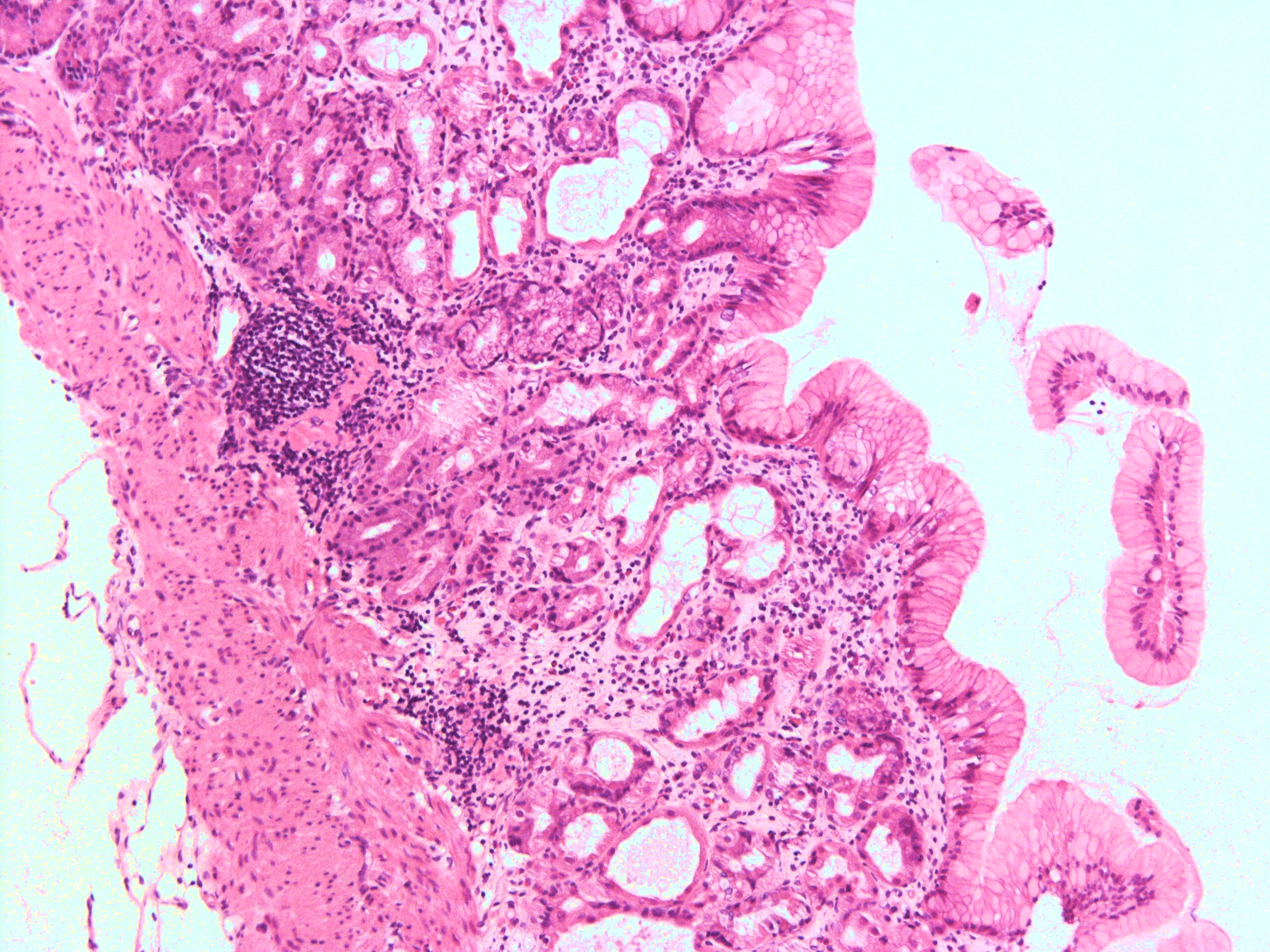
(A, C) Three months after the discontinuation of zolbetuximab, biopsy specimens of the non-inflammatory sites in the gastric corpus and antrum show improvement of inflammatory cell infiltration and regeneration of the glandular epithelium (H&E, ×100). (B, D) Regenerated gastric epithelial cells and glandular cells are positively stained with claudine-18 antibody (34H14L15; abcam, Cambridge, UK).

**A**

**B**

**C**

**D**
